# Heterogeneous Aging Effects on Functional Connectivity in Different Cortical Regions: A Resting-State Functional MRI Study Using Functional Data Analysis

**DOI:** 10.1371/journal.pone.0162028

**Published:** 2016-09-22

**Authors:** Pin-Yu Chen, Jeng-Min Chiou, Ya-Fang Yang, Yu-Ting Chen, Hsin-Long Hsieh, Yu-Ling Chang, Wen-Yih I. Tseng

**Affiliations:** 1 Department of Life Science, National Taiwan University, Taipei, Taiwan; 2 Institute of Medical Device and Imaging, National Taiwan University College of Medicine, Taipei, Taiwan; 3 Institute of Statistical Sciences, Academia Sinica, Taipei, Taiwan; 4 Department of Psychology, National Taiwan University, Taipei, Taiwan; 5 Molecular Imaging Center, National Taiwan University, Taipei, Taiwan; Institute of Psychology, Chinese Academy of Sciences, CHINA

## Abstract

Brain aging is a complex and heterogeneous process characterized by the selective loss and preservation of brain functions. This study examines the normal aging effects on the cerebral cortex by characterizing changes in functional connectivity using resting-state fMRI data. Previous resting-state fMRI studies on normal aging have examined specific networks of the brain, whereas few studies have examined cortical-cortical connectivities across the entire brain. To characterize the effects of normal aging on the cerebral cortex, we proposed the Pearson functional product-moment correlation coefficient for measuring functional connectivity, which has advantages over the traditional correlation coefficient. The distinct patterns of changes in functional connectivity within and among the four cerebral lobes clarified the effects of normal aging on cortical function. Besides, the advantages of the proposed approach over other methods considered were demonstrated through simulation comparisons. The results showed heterogeneous changes in functional connectivity in normal aging. Specifically, the elderly group exhibited enhanced inter-lobe connectivity between the frontal lobe and the other lobes. Inter-lobe connectivity decreased between the temporal and parietal lobes. The results support the frontal aging hypothesis proposed in behavioral and structural MRI studies. In conclusion, functional correlation analysis enables differentiation of changes in functional connectivities and characterizes the heterogeneous aging effects in different cortical regions.

## Introduction

The blood-oxygenation-level-dependent (BOLD) signal measured through fMRI reflects hemodynamic changes resulting from local neural activity [1, 2]. The neural activity interacts with the surrounding vasculature, and the degree of hemodynamic response depends on the dynamic cerebral blood flow, metabolic rate of glucose, and neurovascular coupling [3]. In addition to neuronal loss, studies have shown that the cerebrovascular system is also altered in normal aging under the influence of multiple factors, and such alteration influences the hemodynamic coupling [4, 5]. Therefore, the MRI-derived BOLD signal reflects the spatial pattern of neural and vascular functions and reveals associated changes in the aging brain.

During the resting state, subjects are asked not to perform any explicit cognitive tasks. All spatial patterns of neural activity observed through resting-state fMRI reflect internal activities of the brain [6]. Fluctuations in the BOLD signal can be compared across brain regions [7] by calculating functional connectivity, which indicates correlational strength between the regions [8]. Resting-state fMRI data analysis involves evaluating coherent activity among brain regions and characterizing the functional connectivity of brain networks [9, 10]. Evidence has increasingly clarified that coherent or correlated fluctuation in the resting-state BOLD signal is a steady characteristic of the human brain [11]. Consequently, functional connectivity has been used to reveal intrinsic brain networks and pairwise relations of brain activities [12].

A widely used approach to functional connectivity analysis is to calculate the traditional correlation coefficients (TCC) between a pair of pixels via

$$TCC=\frac{\sum_{i=1}^{N}\left(f_{i}-\mu_{f}\right)(r_{i}-\mu_{r})}{{\left[\sum_{i=1}^{N}{\left(f_{i}-\mu_{f}\right)}^{2}\right]}^{1/2}{\left[\sum_{i=1}^{N}\left(r_{i}-\mu_{r}\right)\right]}^{1/2}},$$ Eq 1

where *f*_*i*_ and *r*_*i*_ are the time-courses (TCs) in given pixels, and *μ*_*f*_ and *μ*_*r*_ are the average values of the TC observations *f*_*i*_’s and *r*_*i*_’s [13, 14]. However, such calculations consider the TC signals as a series of independent signals, overlooking that the TC signals are inter-dependent samples of a continuous function. As a result, each TC is centered on its constant individual mean, and, thus, relative magnitudes between the individual subjects are not relevant, and only the shapes of the signal profiles matter.

It is natural to treat BOLD signal profiles of a brain region as realizations sampled from a stochastic process, treating these measurements as a function of time. Under this sampling frame, the TC signal recordings compose a set of functional data, where each random function corresponds to a particular brain region. Functional data analysis (FDA) applies statistical methods to data sampled from random functions. Following the line of functional data analysis, this study proposes the functional correlation method to measure cortical-cortical functional connectivity.

Using FDA approach to analyzing TC signal recordings has the advantage that it takes the entire signal profile of a cortical region of interest (ROI) as the core unit for the statistical analysis and, thus, automatically takes into account temporal patterns of the signal profiles and correlations between different ROIs. FDA has been extensively applied in such fields as biomedicine, biology, engineering and environmental science. In particular, FDA in functional brain imaging studies was discussed by Tian [15], and techniques for medical applications were reviewed by Sørensen, Goldsmith [16]. FDA methods and applications were systematically reviewed by Ramsay and Silverman [17], Ferraty and Vieu [18], Horváth and Kokoszka [19], Wang, Chiou and Müller [20].

It is worth noting that the BOLD signal profiles of different brain regions of the same subject are mutually dependent. It is a statistical challenge to take into account the within-subject correlations of the random functions of the signal profiles when investigating cortical-cortical connectivities of the entire brain. The set of simultaneously recorded region-specific BOLD signal profiles of subjects intrinsically formed a set of *multivariate* functional data sampled from a set of *multivariate* random functions. To deal with this issue, we used the *multivariate* functional data method, taking the advantage of the additional information of between-ROI correlations within the same subject in the analysis of cortical-cortical connectivities of the brain. The present study aimed to investigate the effects of aging on functional brain connectivity by correlating BOLD signals between the ROIs using functional data analysis.

Previous rsfMRI studies applied the graph theory to explore the change in network properties in the aging brain [21–24]. Here, we used the FDA methods to characterize aging-related changes in resting-state brain activity by calculating cortical-cortical connectivities across the whole brain, instead of focusing on specific networks. Such approach was never reported in the rsfMRI literature. In particular, we performed the simulations to compare the proposed method with other methods of measuring functional connectivity.We examined whether the proposed method distinguishes the effects of aging in large-scale cortical-cortical connectivities across the whole brain.

## Material and Preprocessing

### Participants

Forty young healthy adults and thirty elderly healthy adults were recruited (young group: age = 19–41 years, mean age = 26.2 years, 16 males; elderly group: age = 60–90 years, mean age = 68.6 years, 16 males; all right-handed). The experiment was approved by the Institutional Review Board of the National Taiwan University Hospital. All subjects were apprised of MRI safety concerns and human rights. All subjects provided written informed consent. To ensure cognitive intactness, all subjects in the elderly group were tested on a standardized battery of neuropsychological tests. Elderly subjects with a current or past diagnosis of neurological or psychiatric disorders, substance abuse, or head injury with loss of consciousness were excluded.

### Data acquisition

Structural and functional MRI data were acquired using a 3-Tesla MRI system (Tim Trio, Siemens, Erlangen, Germany) with a 32-channel head coil. Structural MRI included high-resolution T1-weighted imaging and T2-weighted imaging for registration. High-resolution T1-weighted imaging was performed using a three-dimensional magnetization-prepared rapid gradient echo sequence—repetition time (TR)/echo time (TE) = 2000 ms/3 ms, flip angle (FA) = 9°, acquisition matrix = 256 × 192 × 208, and field of view (FOV) = 256 × 192 × 208 mm^3^—yielding an isotropic spatial resolution of 1 mm^3^. T2-weighted imaging was performed using a two-dimensional fast spin-echo sequence: TR/TE = 9422 ms/101 ms, image matrix size = 256 × 256, FOV = 256 × 256 mm^2^, thickness = 3 mm without gap, and slices = 34. During the resting state, subjects were asked to lay still with eyes closed and not to perform explicit cognitive tasks. A two-dimensional gradient-echo planar imaging (GRE-EPI) sequence was used to acquire resting-state fMRI data: TR/TE = 2000 ms/24 ms, FA = 90°, thickness = 3 mm, FOV = 256 x 256 mm^2^, acquisition matrix = 64 x 64, slices = 34, and number of measurements = 180. Resting-state fMRI scan time was approximately 6 minutes.

### Resting-state fMRI and preprocessing

**Fig 1:**
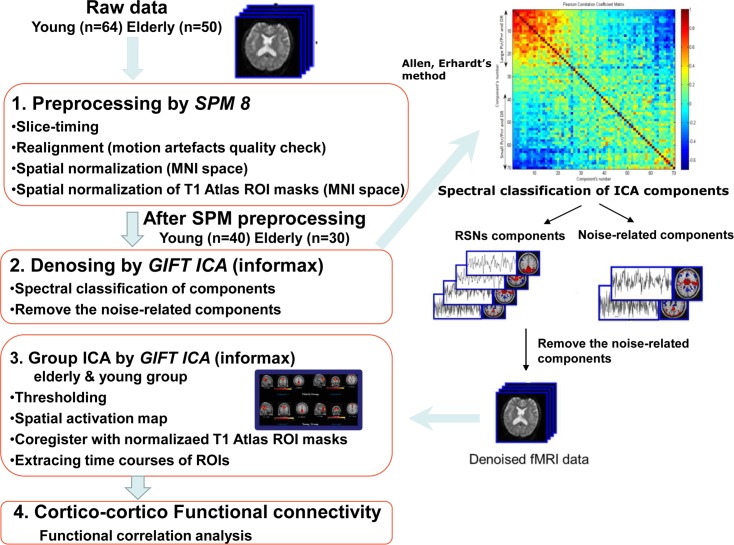
Resting-state fMRI preprocessing.

Resting-state fMRI images were preprocessed using SPM8 (http://www.fil.ion.ucl.ac.uk/spm/, Friston, 2007) and subsequently an in-house independent component analysis (ICA)-based denoising algorithm [25]. Preprocessing proceeded as follows (Fig 1). The first three volumes were discarded to avoid T1 equilibrium effects. The remaining images underwent slice-timing correction, with the middle slice used as the reference frame for correcting acquisition time differences within the volume. The images were then realigned using a rigid-body model. The functional images were registered to the T1-weighted image of each subject. For enhanced normalization, the T1-weighted image was segmented into gray matter, white matter, and cerebrospinal fluid (CSF) on the basis of SPM8’s tissue probability maps (TPMs). A voxel’s TPM represented its probability being the gray matter, white matter, or CSF. The segmentation routine in SPM8 produced spatial normalization parameters by default. After segmentation, the functional images and T1-weighted images were normalized to the standard MNI space by using the normalization parameters and resliced to 2 × 2 × 2 mm^3^ in voxel size. To eliminate low-frequency drift, the functional images were high-pass filtered at a cutoff frequency of 0.01 Hz and smoothed using a Gaussian kernel with a full-width at half-maximum of 6 mm.

The ICA-based denoising method was highly subjected to image quality; it worked well in data with head motion no more than 1 mm in translation and 1 degree in rotation. We obtained these parameters from the preprocessing steps in SPM8. After SPM8 preprocessing, images of 40 out of 64 subjects in the young group and 30 out of 50 subjects in the elderly group satisfied the criteria. The images that did not pass the criteria were excluded from the ICA procedures. The in-house ICA-based denoising algorithm was implemented as follows. It followed Allen and Erhardt’s approach in which group ICA was performed [26]. Group spatial ICA was applied to decompose data sets into 70 maximally independent spatial maps and their corresponding TCs by using the infomax approach, which was repeated 20 times by using GIFTv1.3 (Medical Image Analysis Lab; http://mialab.mrn.org/software/gift/index.html); the results of group spatial ICA were sorted by time course spectral power estimates to determine the RSNs [26]. The spectral power in our data ranged from 0.015 to 0.045 Hz. We used the low frequency to high frequency power ratio of 4 as the cut point to differentiate the RSNs-related from the nuisance signals, such as CSF and physiological noise. Denoising was performed on a subject-by-subject basis by removing the noise-related components from the original BOLD signals.

To explore the cortical regions activated during the resting state, we used ICA to obtain a group activation map before applying our proposed method on the resting-state fMRI data. Group spatial ICA was used to detect commonly revealed RSNs at the group level. The group-level commonly revealed activated regions were our targets for further examination using FDA.

Spatial ICA decomposed the preprocessed resting-state fMRI data sets of the 70 subjects into maximally independent spatial maps and their corresponding TCs by using the infomax approach [27] through GIFTv1.3i (http://mialab.mrn.org/software/gift/). The number of components for decomposition was estimated using minimum description length criteria [28]. The infomax ICA algorithm was repeated 20 times by using a bootstrapping algorithm.

### T1W preprocessing and registering with rsfMRI images

T1 Atlas ‘aparc.a2005s.annot’ was chosen from FreeSurfer (http://surfer.nmr.mgh.harvard.edu/), containing cortical anatomical label information of the whole brain with a total of 162 segmented regions. The group ICA maps and the segmented cortical regions from T1 Atlas ‘aparc.a2005s.annot’ were registered and normalized into the Montreal Neurological Institute (MNI) structural template (http://imaging.mrc-cbu.cam.ac.uk/imaging/Templates) and overlapped to register the anatomical location of the activated regions during the resting-state. Overall, 120 of 162 cortical ROIs were selected from the union of ICA activation maps of the young and elderly groups. Removing limbic ROIs yielded 112 ROIs of the four cortical lobes. The TCs of the 112 ROIs for the young and elderly groups were used as the FDA input. (The ROIs in the T1 Atlas ‘aparc.a2005s.annot’were listed in S2 File.)

## Methods

We define a functional correlation to measure the intensity of the pairwise ROI connectivity based on BOLD signal profiles. The point of view we adopt in the correlation analysis is that each observed BOLD signal profile is a realization of a random function. To define the proposed functional correlation, let the random function *X*_*gk*_(t) denote the measurements of BOLD signals sampled at time *t* in an interval *Ƭ* for the *k*th ROI and the g-*th* group, *k* = 1, …, *K*, and *g* = 1…, *G*. In this study, *K* = 112 for the total number of ROI and *G* = 2 for the total number of groups with *g* being 1 for the young and 2 for the elderly groups.

We propose the *Pearson Functional product-moment Correlation coefficient* (PFCorr) between two random functions *X*_*gk*_ and *X*_*gl*_ as a measure of functional connectivity by

$$\rho_{gkl}^{PFC}=\frac{E(\int_{Ƭ}^{}\left\{X_{gk}(t)-\mu_{gk}\;(t)\right\}\{X_{gl}\;(t)-\mu_{gl}(t)\}dt)}{{\left[E(\int_{Ƭ}{\left\{X_{gk}(t)-\mu_{gk}(t)\right\}}^{2}dt)\right]}^{1/2}{\left[E(\int_{Ƭ}{\left\{X_{gl}(t)-\mu_{gl}(t)\right\}}^{2}dt)\right]}^{1/2}},$$ Eq 2

where *μ*_*gk*_(*t*) = E{*X*_*gk*_(*t*)} is the mean function of *X*_*gk*_(*t*). The PFCorr represents the cross-correlation between two random functions. Analogous to the classical cross-correlation coefficient that measures the degree of association between two random variables in Euclidean space, the proposed PFCorr measures the strength of association between two random functions in a square-integrable Hilbert space.

The sample version of PFCorr can be obtained by the numerical approximations to the means of the integration in Eq 2. However, the observed BOLD signals can be contaminated by measurement errors and the random functions are not observed directly. Furthermore, the BOLD signals from different brain regions of an individual subject are highly dependent. We suggest a model-based approach to the estimation of PFCorr using the multivariate functional principal component analysis [29]. This model-based approach not only takes into account measurement errors of the signals but also borrows the strength of within-subject correlations among different brain regions.

### Review of multivariate functional principal component analysis

Let {*X*_*gki*_(*t*); *i* = 1,…,*n*_*g*_} be a sample of independently and identically distributed random functions with respect to the subject index *i*, which are sampled from the distribution of *X*_*gk*_(*t*). To take into account the within-subject correlations between ROIs, let *X*_*gi*_(*t*) = (*X*_*g*1*i*_(*t*),…,*X*_*gKi*_(*t*))^T^ be a vector of the multivariate random functions with the mean function *μ*_*g*_(*t*) = (*μ*_*g*1_(*t*),…,*μ*_*gK*_(*t*)) whose *k*th entry *μ*_*gk*_(*t*) = E{*X*_*gk*_(*t*)}, and the multivariate covariance function *G*_*g*_(*s*,*t*) = (*G*_*gkl*_(*s*,*t*), 1 ≤ *k*,*l* ≤ *K*) whose (*k*, *l*) entry is *G*_*gkl*_(*s*,*t*) = *cov*(*X*_*gk*_(*s*),*X*_*gl*_(*t*)), which is the auto-covariance function of *X*_*gk*_(t) when *k = l* and the cross-covariance function between *X*_*gk*_(*t*) and *X*_*gl*_(*t*) when *k* ≠ *l*. In consideration of possible measurement errors in practice, we consider the model *Y*_*gij*_ = *X*_*gi*_(*t*_*j*_) + *ϵ*_*gij*_, where *ϵ*_*gij*_ = (*ϵ*_*g*1*ij*_,…,*ϵ*_*gKij*_)^T^ are independently and identically distributed with respect to *i* and *j* with mean zero and variance $\sigma_{g}^{2}={\left(\sigma_{g1}^{2},\ldots ,\sigma_{gK}^{2}\right)}^{T}.$ Here, j = 1,…,J, J = 177.

Using the multivariate functional principal component (mFPC) model [29] for the multivariate random function *X*_*gi*_(*t*) by

$$X_{gi}(t)=\mu_{g}(t)+\sum_{r=1}^{\infty }\xi_{gri}(D_{g}\varphi_{gr})(t),$$ Eq 3

where *D*_*g*_(*t*) = *diag*(*v*_*g*1_(*t*)^1/2^,…,*v*_*gK*_(*t*)^1/2^) is a diagonal matrix with elements *v*_*gk*_(*t*) = *G*_*gkk*_(*t*,*t*) the variance function of *X*_*gk*_,*φ*_*gr*_(*t*) = (*φ*_*g*1*r*_(*t*),…,*φ*_*gKr*_(*t*))^T^ is the vector of eigenfunctions satisfying $\sum_{k=1}^{K}\langle \varphi_{gkr},\varphi_{gkr\prime }\rangle =\sum_{k=1}^{K}\int_{T}^{}\varphi_{gkr}(t)\varphi_{gkr^{\prime }}(t)dt=\delta_{rr\prime }$ with $\delta_{rr^{\prime }}=1$ for *r = r'* and 0 otherwise. The random coefficient $\xi_{gri}=\sum_{k=1}^{K}\langle v_{gk}^{-1/2}(X_{gki}-\mu_{gk}),\varphi_{gkr}\rangle$ has the mean of zero and the variance *λ*_*gr*_, in non-ascending order in *r*. The realizations of the random coefficient *ξ*_*gri*_ are called the *r*th mFPC scores for subject *i*. We assume the sequence of the variances {*λ*_*gr*_; *r* ≥ 1} decays rapidly so that the sum of the infinite series in Eq 4 can be well approximated by the sum of the first *L*_*g*_ terms. Therefore, we have a truncated representation for the multivariate random functions,

$${\tilde{X}}_{gi}(t)=\mu_{g}(t)+\sum_{r=1}^{L_{g}}\xi_{gri}(D_{g}\varphi_{gr})(t),$$ Eq 4

The estimation of PFCorr is based on the model Eq 4. In practice, as in the conventional multivariate analysis, the number of components *L*_*g*_ can be chosen using the criterion of the proportion of total variance explained by the leading components.

#### Examination of the gender effect

Gender effect has drawn attention in the literature [30–34]. Since there were no participants aged between 20 and 40 in our study, we elaborated the gender effect at the young/elderly group level. We considered a two-sample test of the mean BOLD signal profiles between male and female within each group. Instead of directly using the BOLD signal profiles for two sample test of the means, we made use of the mFPC scores as the proxies of the BOLD signal profiles to test the overall gender effect on the whole brain at each group level. Thus, testing the gender effects on equality of two mean functions reduced to a classical multivariate two-sample problem through the mFPC analysis. Here, we applied the most widely used test of the means of two random vectors by the classical Hotelling's *T*^2^ test under the multivariate normality assumption. Since the two-sample *T*^2^ test became slightly conservative when the normality assumption was violated [35], we also implemented the hypothesis test based on the bootstrap resampling method without any distributional assumption by the resampling procedure described in S1 File. Tables A and B in S1 File summarize the outcome of the tests, with the corresponding value *L* such that the explained variance is at least 80%. It fails to reject the null hypothesis in the elderly group while the p-value is relatively small when *L* = 8 in the young group, which suggests that gender might signify certain degree of differences in resting state fMRI in some brain regions for young subjects, even though it is not smaller than 0.05.

#### Estimation of the function correlation

Under the mFPC model in Eq 4, PFCorr in Eq 2 can be obtained based on the following expression,

$${\tilde{\rho }}_{gkl}^{PFCm}=\frac{\sum_{r=1}^{L_{g}}\lambda_{gr}\langle v_{gk}^{1/2}\varphi_{gkr},\;v_{gl}^{1/2}\varphi_{glr}\rangle }{{\left\{\sum_{r=1}^{L_{g}}\lambda_{gr}{\left\|v_{gk}^{1/2}\varphi_{gkr}\right\|}^{2}\right\}}^{1/2}{\left\{\sum_{r=1}^{L_{g}}\lambda_{gr}{\left\|v_{gl}^{1/2}\varphi_{glr}\right\|}^{2}\right\}}^{1/2}}$$ Eq 5

Here, the parameters *λ*_*gr*_, *V*_*gk*_ and *φ*_*gkr*_ in Eq 5 are unknown and the value *L*_*g*_ is to be determined. The nonparametric estimation procedure of the parameter estimates is based on the observations *Y*_*gij*_, which we refer to Chiou, Chen and Yang [29] for the details. These lead to the predicted individual BOLD signal trajectory

$${\hat{X}}_{gi}(t)={\hat{\mu }}_{g}(t)+\sum_{r=1}^{L_{g}}{\hat{\xi }}_{gri}({\hat{D}}_{gi}{\hat{\varphi }}_{gr})(t),$$ Eq 6

and a sample version of PFCorr Eq 5,

$$r_{gkl}^{PFCm}=\frac{\sum_{r=1}^{L_{g}}{\hat{\lambda }}_{gr}^{}\langle {\hat{v}}_{gk}^{1/2}{\hat{\varphi }}_{gkr},{\hat{v}}_{gl}^{1/2}{\hat{\varphi }}_{glr}\rangle }{{\left\{\sum_{r=1}^{L_{g}}{\hat{\lambda }}_{gr}^{}{\left\|{{\hat{v}}_{gk}^{1/2}}_{}^{}{\hat{\varphi }}_{gkr}\right\|}^{2}\right\}}^{1/2}{\left\{\sum_{r=1}^{L_{g}}{\hat{\lambda }}_{gr}^{}{\left\|{{\hat{v}}_{gl}^{1/2}}_{}^{}{\hat{\varphi }}_{glr}\right\|}^{2}\right\}}^{1/2}}.$$ Eq 7

${\hat{\mu }}_{g},\;{\hat{D}}_{g},\;{\hat{\varphi }}_{gr},\;{\hat{\lambda }}_{gr}\;and\;{\hat{\xi }}_{gri}$ represent the estimates of the corresponding terms and the number of components *L*_*g*_ is chosen data-adaptively by

$$L_{g}=arg\;\min_{M}\left\{\sum_{r=1}^{M}{\hat{\lambda }}_{gr}/\sum_{r=1}^{\infty }{\hat{\lambda }}_{gr}1_{\left\{{\hat{\lambda }}_{gr}>0\right\}}>\delta \right\}$$ Eq 8

for a fixed 0 < δ < 1, indicating that the *L*_*g*_ leading components explain at least 100δ% of the total variance of the multivariate functional data. Here, we choose δ = 0.90, which works reasonably well in this study.

#### Inference for the aging effects on functional connectivity

To investigate the normal aging effect on functional connectivity, we evaluate the statistical significance of the paired PFCorr differences between the young and the elderly groups by testing the hypotheses below.

$$H_{0}^{\left(k,l\right)}:\;\rho_{1kl}^{PFC}=\rho_{2kl}^{PFC}\;vs.\;H_{a}^{\left(k,l\right)}:\;\rho_{1kl}^{PFC}\neq \rho_{2kl}^{PFC}\;,\;1\leq k\leq l\leq K.$$ Eq 9

The null hypothesis indicates the functional correlations of the *(k*,*l)* ROI pair between the young and the elderly group are not significantly different. The test statistics is constructed based on the estimate of PFCorr. We propose to test the hypotheses in Eq 9 based on the bootstrap resampling method [36] to obtain the null distribution of $\left(r_{2kl}^{PFCm}-r_{1kl}^{PFCm}\right)$. The bootstrap testing procedure is summarized as follows.

1. Combine the observations of the young and the elderly groups to form *Z*_*ij*_ for *i* = 1, …, *n*, where *n* = *n*_1_ + *n*_2_.
2. Perform the mFPC analysis on *Z*_*ij*_ and obtain the model component estimates $\hat{\mu }$, ${\hat{\xi }}_{ri}$, $\hat{D}$, and ${\hat{\varphi }}_{r}$ of the *μ*, *ξ*_*ri*_. *D*, and *φ*_*r*_ in Eq 4. Let ${\hat{\xi }}_{i}={\left({\hat{\xi }}_{1i},\ldots ,{\hat{\xi }}_{Mi}\right)}^{T}$, where *M* is chosen by the criterion of the proportion of total variance explained as in Eq 7.
3. Resample $\left\{{\hat{\xi }}_{i},\;Z_{ij};j=1,\ldots ,J\right\}$ by the subject indices *i* ∈ {1,…,*n*} with replacement *n* times to form the bootstrap samples $\left\{{\hat{\xi }}_{i}^{b},\;Z_{ij}^{b};j=1,\ldots ,j\right\}$ of size *n*, where *b* is the replicate index of the bootstrap samples, *b* = 1, …., *B*. We set *B* = 1000 as the total number of bootstrap samples.
4. To form the bootstrap sample for each group, take the *n*_*g*_ observations in the order of *i* from $\left\{{\hat{\xi }}_{i}^{b}\right\}$ and $\left\{Z_{ij}^{b}\right\}$ to form $\left\{{\hat{\xi }}_{gi}^{b}\right\}$ and $\left\{Y_{gij}^{b}\right\}$, *g* = 1, 2. Obtain the estimates ${\hat{\lambda }}_{gr}^{b}$ by the sample variance of $\left\{{\hat{\xi }}_{i}^{b}\right\}$ and the variance function $v_{gk}^{b}$(t) using the local polynomial smoothing on the squared residuals of $Y_{gkij}^{b}$ (see [37]).
5. Calculate the function correlations $r_{gkl}^{PFCm,b}$ as in Eq 7 for each of the bootstrap samples, *b* = 1, …, *B*, by
  $$r_{gkl}^{PFCm,b}=\frac{\sum_{r=1}^{L_{g}}{\hat{\lambda }}_{gr}^{b}\langle {\left({\hat{v}}_{gk}^{b}\right)}^{1/2}{\hat{\varphi }}_{gkr},\;{\left({\hat{v}}_{gl}^{b}\right)}^{1/2}{\hat{\varphi }}_{glr}\rangle }{{\left\{\sum_{r=1}^{L_{g}}{\hat{\lambda }}_{gr}^{b}{\left\|{\left({(\hat{v}}_{gk}^{b}\right)}^{1/2}{\hat{\varphi }}_{gkr}\right\|}^{2}\right\}}^{1/2}{\left\{\sum_{r=1}^{L_{g}}{\hat{\lambda }}_{gr}^{b}{\left\|{{(\hat{v}}_{gl}^{b})}^{1/2}{\hat{\varphi }}_{glr}\right\|}^{2}\right\}}^{1/2}}.$$
6. For each (*k*, *l*) pair, 1 ≤ *k*,*l* ≤ *K*, calculate the group difference in functional correlations, $U_{kl}^{b}=(r_{2kl}^{PFCm,b}-r_{1kl}^{PFCm,b})$, and compute the unadjusted *p*-value under null hypothesis *H*_0_ by $P_{kl}=B^{-1}\sum_{b=1}^{B}1(\left|U_{kl}^{b}|>|U_{kl}\right|)$, where $U_{kl}^{}=(r_{1kl}^{PFCm}-r_{2kl}^{PFCm})$ and $1(\left|U_{kl}^{b}|>|U_{kl}\right|)$ is the indicator function with the value 1 when $\left(\left|U_{kl}^{b}|>|U_{kl}\right|\right)$ and 0 otherwise.
7. Order the *p*-value {*P*_*kl*_; 1 ≤ *k*,*l* ≤ *K*} associate with $\left\{H_{0}^{\left(k,l\right)};1\leq k,l\leq K\right\}$ denoted as *P*_(1)_ ≤ *P*_(2)_ ≤ ⋯ ≤ *P*_(*m*)_ with the associated hypotheses *H*_0(1)_,*H*_0(2)_,…,*H*_0(*m*)_, where *H*_0(*r*)_ is the hypothesis corresponding to some $H_{0}^{\left(k,l\right)}:\;\rho_{1kl}^{PFC}=\rho_{2kl}^{PFC}$ for some pair (*k*, *l*), *k* ≠ *l*, 1 ≤ *r* ≤ *m*, and *m* = *K*(*K* − 1)/2 is the amount of hypotheses to be tested. Reject all *H*_0(*q*)_ for *q* = 1,…,*Q*, with $Q=q({\hat{m}}_{0})=max\{q:P_{\left(q\right)}\leq \frac{q}{{\hat{m}}_{0}}\alpha {\left\lfloor \sum_{q=1}^{m}q^{-1}\right\rfloor }^{-1}|{\hat{m}}_{0}\}$, at the significant level α = 0.05, where ${\hat{m}}_{0}=max\{L:L\leq m-q(L)\}$.

**Fig 2:**
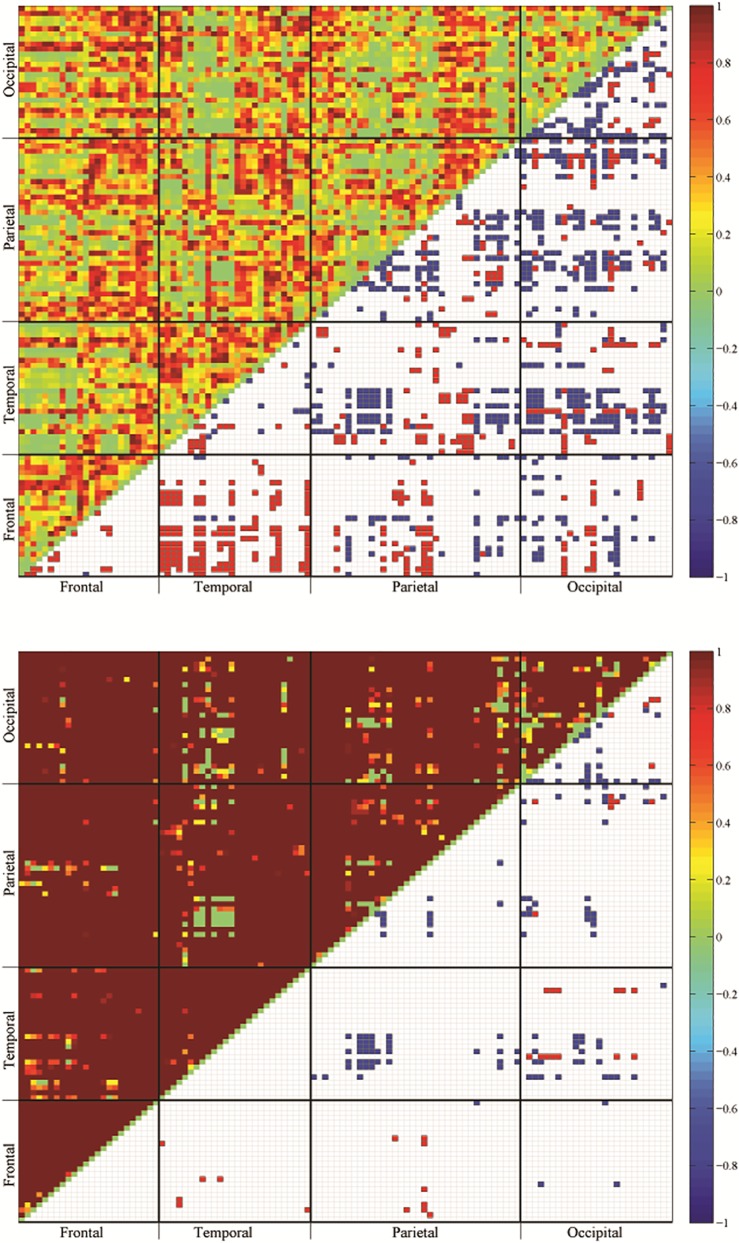
The uncorrected (top panel) and corrected (bottom) *p*-values (upper triangular matrix) of testing the paired difference of the Pearson Functional Correlations between the young and the elderly groups, $r_{2kl}^{{PFC}_{m}}-r_{1kl}^{{PFC}_{m}}$ (elderly–young). The ROI pairs with significant difference are marked in blue for negative values and in red for positive values in the lower triangular matrix.

The final step of the bootstrap test procedure is a modified Benjamini-Hochberg procedure, taking into account the dependency of multiple hypotheses and controlling the FDR (False Discovery Rate) [38]. Fig 2 illustrates the uncorrected (top panel) and the corrected (bottom) *p*-values for the pairwise hypothesis test of the mean functions. By comparison, the uncorrected *p* -values tend to conclude many insignificant pairs, while the corrected *p* -values identify 173 ROI pairs that are significantly different.

## Results

**Fig 3:**
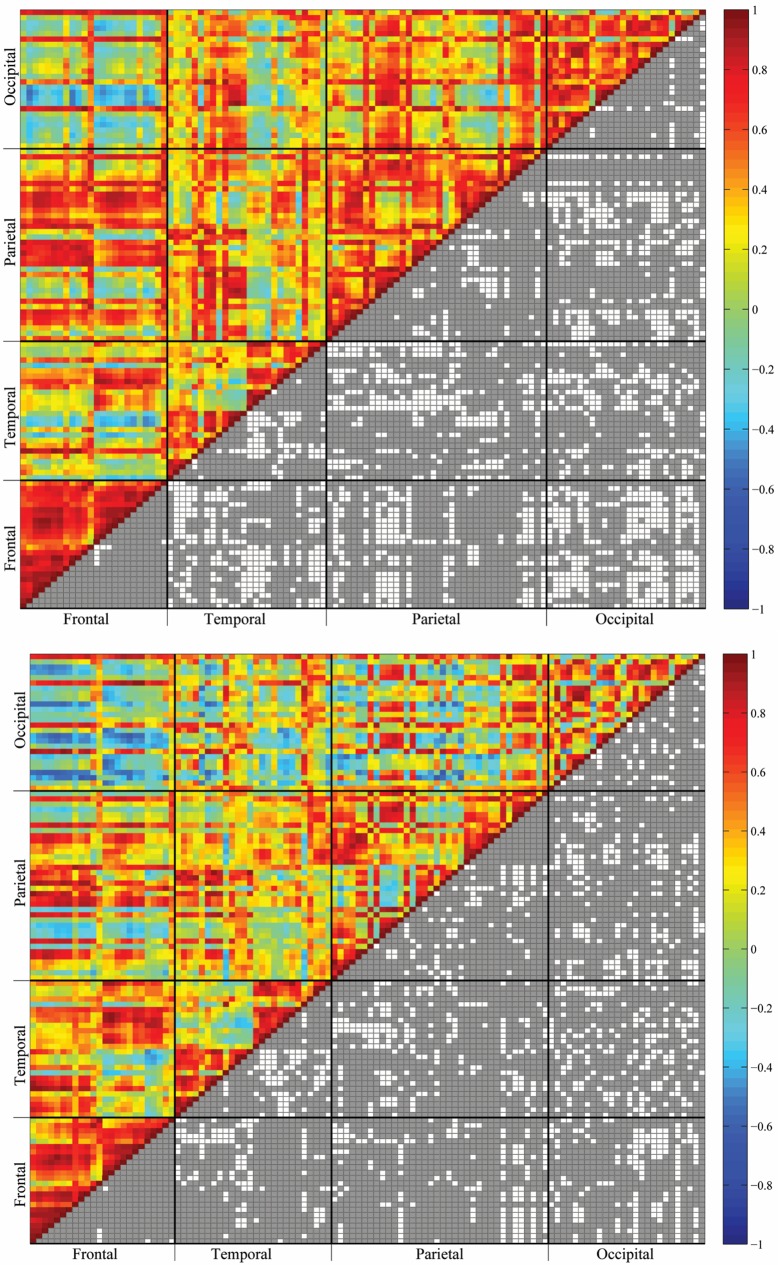
Cortical-Cortical Connectivity (CCC) matrices for the young (top) and the elderly (bottom) groups. The colors on the upper triangular matrices indicate the PFCorr connectivity intensity of the ROI pairs. The gray spots on the lower triangular matrices indicate ROI pairs with statistically significant nonzero correlations.

Among the 112 cortical ROIs, 6216 functional correlations were observed. The strengths of the functional correlations were presented in the Cortical-Cortical Connectivity (CCC) matrix. Fig 3 shows the matrices of the CCC for the young and elderly groups. These two matrices can reveal whether the CCC across the cortex is homogeneous or heterogeneous. In other words, the patterns of the CCC among and within the four cortical lobes can reveal the aging characteristics of the cortex. The most evident finding was the heterogeneous patterns of both inter-lobe and intra-lobe connectivities. Compared with the young group, the elderly group generally exhibited decreased functional correlations in both inter-lobe and intra-lobe connectivities among the posterior lobes, namely the temporal, parietal, and occipital lobes.

**Fig 4:**
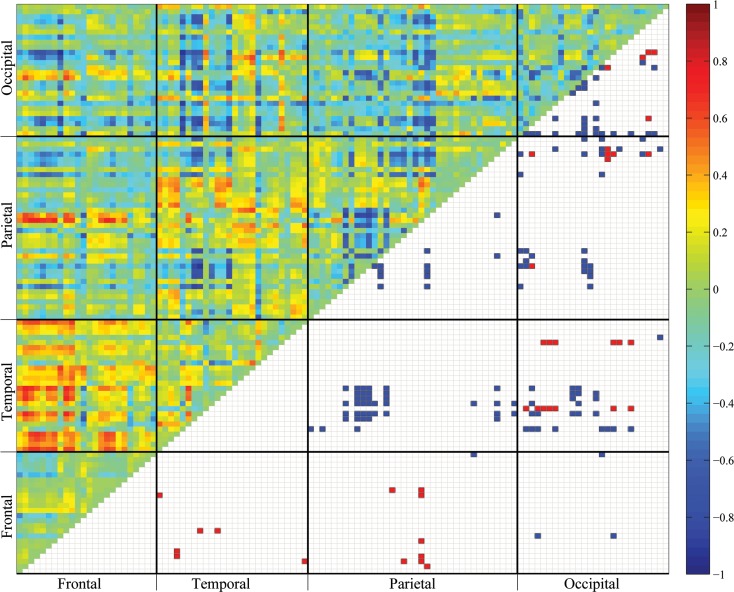
Paired differences of the brain connectivity matrices between the young and the elderly groups ($r_{2kl}^{{PFC}_{m}}-r_{1kl}^{{PFC}_{m}}$). The colors on the upper triangular matrix indicate the PFCorr difference of the ROI pairs. The significantly different ROI pairs are shown on the lower triangular matrix, with negative values marked in blue and positive values marked in red.

To investigate effects of normal aging on functional connectivity, we evaluated the differences in the CCC matrix by subtracting PFCorr of the young group from those of the elderly group. Significant group differences were classified into two aging effects: positive and negative (Fig 4). A positive aging effect indicated that the functional connectivity strength increased with age, whereas a negative aging effect indicated that the strength of functional connectivity decreased with age. Of 6216 correlations, 173 showed a significant aging effect; 39 exhibited positive and 134 exhibited negative effects.

Because 50% of the differences in the functional correlation coefficients with significant aging effects exceeded 0.45, we selected 85 connectivities with absolute differences exceeding 0.45 for further analysis. These selected connectivities belonged to the intra-lobe connectivities of the occipital lobes and the inter-lobe connectivities between the frontal and temporal lobes, between the temporal and occipital lobes, between the temporal and parietal lobes, and between the parietal and occipital lobes. By contrast, the connectivities between the frontal and parietal lobes and those between the frontal and occipital lobes showed no evident aging effects; all differences were less than 0.45.

Four types of aging-related changes were observed in the selected connectivities: two positive and two negative aging effects. In the first type of the positive aging effect, the connectivity revealed negative correlations in the young group but positive correlations in the elderly group. In the second type, the connectivity exhibited positive correlations in the young group and stronger positive correlations in the elderly group. In the first type of the negative aging effect, the connectivity revealed positive correlations in the young group and less positive correlations in the elderly group. In the second type, the connectivity showed positive correlations in the young group but negative correlations in the elderly group. No positive aging effect showed negative correlations in the young group and weaker negative correlations in the elderly group. Similarly, no negative aging effect showed negative correlations in the young group and stronger negative correlations in the elderly group.

**Fig 5:**
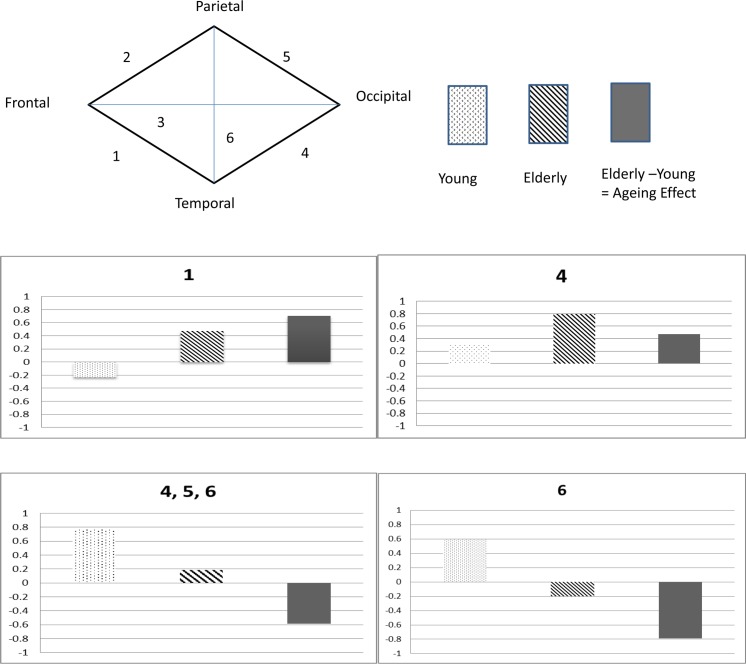
The distinguished normal ageing effects across the cortical lobes. E: elderly group; Y: young group; E-Y: ageing effects (elderly group subtracts young group). Connectivity 1 denotes the connectivity from the frontal lobe to posterior lobes; Connectivity 4, 5, 6 are the interlobe connectivities among the posterior lobes: Connectivity 4 connects the temporal and occipital lobes, Connectivity 5 connects the parietal and occipital lobes, and Connectivity 6 connects the temporal and parietal lobes.

The spatial patterns of the aging effect can be divided into two main groups: the inter-lobe connectivity of the frontal lobe (Connectivity 1 in Fig 5) and the inter-lobe connectivities among the posterior lobes (Connectivities 4, 5, and 6 in Fig 5). The inter-lobe connectivities of the frontal lobe with a positive aging effect were those between the frontal and temporal lobes, which showed negative correlations in the young group but positive correlations in the elderly group (Connectivity 1 in Fig 5). The inter-lobe connectivities among the posterior lobes with a positive aging effect were those between the temporal and occipital lobes, which exhibited positive correlations in the young group and strong positive correlations in the elderly group (Connectivity 4 in Fig 5). Only the inter-lobe connectivities among the posterior lobes showed a negative aging effect: the inter-lobe connectivities between the temporal and occipital lobes, between the parietal and occipital lobes, and between the temporal and parietal lobes, all of which revealed positive correlations in the young group and weaker positive correlations in the elderly group (Connectivities 4, 5, and 6 in Fig 5).The inter-lobe connectivities between the temporal and parietal lobes showed another type of negative aging effect in which the connectivities exhibited positive correlations in the young group but negative correlations in the elderly group (Connectivity 6 in Fig 5).

**Fig 6:**
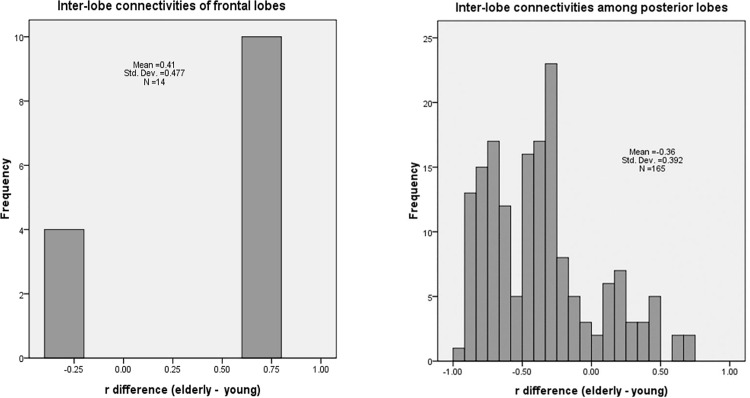
Histograms of the normal aging effects on inter-lobe functional connectivities. The distribution curves indicate that inter-lobe connectivities of the frontal lobe tend to increase with age, whereas those among posterior lobes tend to decrease with age.

Fig 6 shows the significant aging effects of inter-lobe connectivities of the frontal and posterior lobes. Positive and negative aging effects on the inter-lobe functional connectivities were observed. The inter-lobe functional connectivity of the frontal lobe showed a positive aging effect, whereas that of the posterior lobes showed positive and negative aging effects. The inter-lobe functional connectivity between the temporal and occipital lobes showed a positive aging effect. The inter-lobe functional connectivities of the posterior lobes, particularly the connectivities between the temporal and parietal lobes, exhibited a dominant negative aging effect.

### Comparison via simulation study

We define the pairwise Pearson functional correlation Eq 2 using the functional data method, taking TC signal profiles as samples of random functions. The multivariate FPCA approach to the estimate of the functional correlation. Eq 2 further takes the advantages of within-subject correlations of the multivariate random functions corresponding to signal profiles of different brain regions. We compare the proposed method with other estimations, the naïve functional correlation estimate (PFCo) and PFCorr estimate based on univariate FPCA (PFCu). In addition, we compare the methods with the traditional correlation coefficient (TCC) in Eq 1 as mentioned in the Introduction.

- *Native functional correlation estimation* (PFCo) In estimation of the functional correlation PFCorr in Eq 2, a possible naïve estimate $r_{0}^{PFC}$ can be obtained by replacing *X*_*gki*_ with observations {*Y*_*gkij*_}, coupled with numerical integrations. In contrast to the model-based estimate *r*^*PFCm*^ in Eq 6, this naïve estimate does not consider random measurement errors, and may not work well when observations are sparsely sampled in the time points.
- *Pearson product-moment correlation coefficient estimation based on univariate FPCA (PFCu)* In contrast to the mFPCA-based method to estimate PFCorr, it is also possible to estimate the proposed functional correlation by the conventional univariate FPCA (uFPCA) method to express the individual random functions. This approach does not make use of the dependent information between the ROIs within the same subject.
- *Traditional Correlation Coefficient (TCC)* Here, we take the average of traditional correlation coefficients over all subjects as mentioned in Eq 1 in the Introduction as the correlation measure (TCC). TCC is defined in the way that individual profile trajectories are centering on their averages of the individual trajectories, and, thus, relative magnitudes between the profiles are not relevant and only the shapes matters.

We perform simulation comparisons to examine the finite sample performance of the proposed estimate *r*^*PFCm*^, and the estimates $r_{0}^{PFC}$, *r*^*PFCu*^, and *r*^*TCC*^ as defined above. For simulation comparisons, we set *K* = 2, *G* = 1 and generate the observations based on the mFPC model Eq 4 with the number of components *L*_*g*_ = 20. The recording time points *t*_*j*_ are equally spaced on [0,1] with *m* = 51. We generate 200 simulation replicates, with each simulation dataset of size *n* = 100. We set *μ*(*t*) = (*t* − 2.25, cos(*t*))^T^ and *v(t)* $={\left(1.25,\sqrt{t}+5\right)}^{T}$ and the measurement error *ϵ*_*kij*_ following a normal distribution with mean zero and variance $\sigma_{k}^{2}$, where $\sigma_{1}^{2}$ = 1 and $\sigma_{2}^{2}$ = 4. We construct the correlation function C(s,t) = {*C*_*kl*_(*s*,*t*); *k*,*l* = 1,2} by Bessel correlation function of the first kind and Matérn correlation function with parameter (1, 1) in Scenario 1 and (0.8, 0.75) in Scenario 2 [29]. The mFPC scores {*ξ*_*ri*_} are generated from N (0, *λ*_*r*_), where *λ*_*r*_ is the eigenvalue of *C*. Then, we obtain the observations $Y_{i}(t_{j})=X_{i}(t_{j})+\epsilon_{i}=\sum_{r=1}^{20}\xi_{ri}\{D\Phi_{r}\}(t_{j})+\epsilon_{ij},\;for\;t=t_{j}$, where X(*t*_*j*_) = (*X*_1_(*t*_*j*_),*X*_2_(*t*_*j*_))^T^, $D(t_{j})=v^{\frac{1}{2}}(t_{j})$, and *ϵ*_*ij*_ = (*ϵ*_1*ij*_,*ϵ*_2*ij*_)^*T*^.

The simulation results, summarized in Table 1 via the sample bias, standard error, and the mean square errors, indicate that the proposed method outperforms the others. The proposed method performs better than PFCo that does not take into account of random measurement errors. It performs better than PFCu as our method takes the advantages of within-subject correlations of the multivariate random functions corresponding to the signal profiles of different brain regions. The TCC does not perform well since the individually centered functional correlation focuses on the similarity of profile shapes and ignores the relative magnitudes between the signal profiles.

**Table 1:** Performance comparison of the estimated functional correlations based on bias, standard error and mean square error for Scenario 1 and Scenario 2.

	Scenario 1				Scenario 2			
	*r*^*PFCm*^	*r*^*TCC*^	*r*^*PFCu*^	$r_{0}^{PFC}$	*r*^*PFCm*^	*r*^*TCC*^	*r*^*PFCu*^	$r_{0}^{PFC}$
**bias**	**0.014**	**-0.556**	**0.300**	**-0.245**	**-0.029**	**0.097**	**0.037**	**0.128**
**stderr(× 10^−3^)**	**2.715**	**2.067**	**2.234**	**2.138**	**1.786**	**1.670**	**4.811**	**1.900**
**MSE**	**0.002**	**0.310**	**0.091**	**0.061**	**0.002**	**0.010**	**0.006**	**0.017**

**Fig 7:**
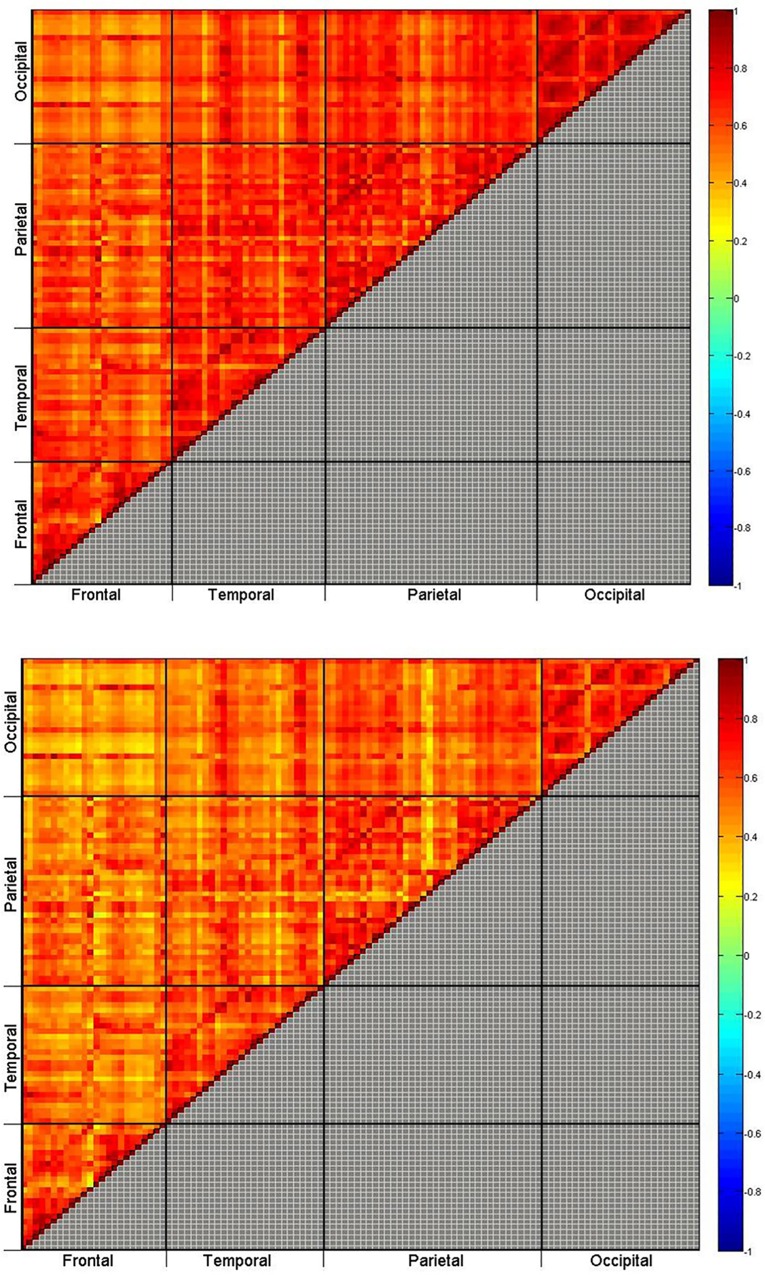
Cortical-Cortical Connectivity (CCC) matrices for the young (top) and the elderly (bottom) groups. The colors on the upper triangular matrices indicate the average individual-centered functional connectivity intensity of the ROI pairs. The gray square elements on the lower triangular matrices indicate ROI pairs with statistically significant nonzero correlations.

Besides applying TCC to the resting-state fMRI data, we obtain the CCC matrices of all ROI pairs for the young and the elderly groups as shown in Fig 7. The correlations of all ROI pairs are all positive and larger than 0.4 in both groups, which appears to be unusual. We conclude that the proposed pairwise Pearson functional correlation using mFPCA as estimate provides a useful correlation measure for the strength of functional connectivity.

## Discussion and Conclusion

To our knowledge, this is the first report on the effects of aging on cortical-cortical functional connectivity. The effects were studied using novel PFCorr statistics-based functional connectivity definitions. Our results showed that the connectivities between the frontal and posterior lobes increased in the elderly group, particularly the connectivity between the frontal and temporal lobe. By contrast, the inter-lobe connectivities among the posterior lobes decreased in the elderly group. The connectivity between the temporal and parietal lobes displayed the strongest negative aging effect. These findings suggest that the aging effect on cortical-cortical functional connectivity is heterogeneous across the lobes.

The essential question regarding the effects of aging on the brain is whether the aging patterns are heterogeneous across the lobes. From MRI data, scholars have reached a consensus on the aging effect that the effect of aging on the cerebral cortex and white matter is heterogeneous [39–41]. These studies examined the structural changes of the gray and white matter by using T1-weighted and diffusion tensor MRI images, respectively. The structure of the cerebral cortex gradually degenerates with age but those of the lobes degenerate at different rates. Moreover, age-related microstructural changes in the white matter vary regionally in the brain. Such heterogeneous characteristics in both the gray and white matter may reflect specific histological changes in the neuronal structures. The present study further examined resting-state cortical-cortical functional connectivity to investigate whether the heterogeneous characteristics are also exhibited by the dynamic cortical connection; the results demonstrated that the aging effect was heterogeneous across the cortical lobes.

Researchers have proposed brain plasticity and compensation to explain the changes in normal aging [5, 42]. According to the compensatory hypothesis, during a cognitive task, elderly people express more activities in certain brain regions than young people do [43]. Many studies have observed that elderly people express more activities in the prefrontal cortex (PFC) [5, 42] and have suggested a compensatory role of the PFC in maintaining normal cognitive functioning. These findings affirm the frontal-aging hypothesis, which states that elderly adults tend to have strong activities in the PFC during task conditions to compensate for reduced activities in other brain regions. Further research can elucidate whether the compensatory aging effect of the PFC also occurs during the resting state of the brain. By demonstrating increased inter-lobe connectivity between the frontal and temporal lobes, our resting-state findings support the compensatory role of the frontal lobe.

The present study demonstrated that the aging effects on inter-lobe functional connectivity can be classified into two main effects: namely the positive (increased) aging effect and the negative (decreased) aging effect. The aging patterns of the frontal and posterior lobes substantially differ. Inter-lobe functional connectivity among the posterior lobes showed a predominantly negative aging effect. By contrast, inter-lobe functional connectivity between the frontal and posterior lobes presented a predominantly positive aging effect, possibly because of the enhanced connectivity of the frontal lobe necessary for maintaining normal brain function in elderly adults. Our finding demonstrated that in the absence of a specific cognitive function, the frontal lobe exhibited strong dynamic cortical-cortical connections. In other words, the age-related changes in resting-state connectivity support the frontal-aging hypothesis.

This study has some limitations. First, this was a cross-sectional comparison study between two age groups. A longitudinal study observing life-long aging effects on the same subjects will be more accurate; however, performing such a study is difficult. Second, this study focused on the functional connectivity of only the cerebral cortex. The aging effects on the functional connectivity of the limbic system and subcortical regions were not studied. Third, given the limited sample size (70 in total) we can only divide the subjects into two groups, i.e. 40 subjects in the young group and 30 subjects in the old group, to achieve statistical power. Future studies could explore a division into a greater number of age groups by acquiring much larger datasets across the lifespan; this could give us greater resolution to identify differences between specific ages. Fourth, our data includes elderly brains which were atrophy. The normalization of the atrophy brain to the template has been a technical issue in the neuroimaging filed. The normalization process mainly relies on the segmentation and spatial normalization via different algorithms. SPM, DARTEL, and FSL tools are the most frequently used. The previous comparative study indicated that the unified segmentation/normalization of SPM8 revealed the largest age-related differences and may overestimate the aging effect on the brain volume [44]. This will lead to increased uncertainty of the BOLD signal extracted from the data of the elderly group, and of the resulting functional connectivity. In this regard, our results may not be the ground truth under the influence of such issue.

In conclusion, we propose a novel functional correlation approach for measuring cortical-cortical functional connectivity. Using this approach, we characterized the heterogeneous aging effects on functional connectivities of the cerebral cortex. Given the characteristic patterns in a normal aging brain, the proposed approach is potentially useful for investigating cortical-cortical functional connectivities in age-associated brain diseases, such as Alzheimer's disease and Parkinson's disease.

## Supporting Information

S1 File Estimation of Gender Effect. (PDF)

S2 File ROI List of T1 Atlas. (PDF)

S3 File Dataset. (RAR)

S4 File Codepackage. (ZIP)
