# Supplementary material for: Heterogeneous Aging Effects on Functional Connectivity in Different Cortical Regions: A Resting-State Functional MRI Study Using Functional Data Analysis
Source: PLoS One. 2016 Sep 22;11(9):e0162028. doi: 10.1371/journal.pone.0162028 (PMC5033468; doi:10.1371/journal.pone.0162028)
Supplement: S1 File — (PDF) [file pone.0162028.s001.pdf]

Supporting Information

S1 File. Estimation of Gender Effect

To explain the bootstrap testing procedure, let $\xi_i = (\xi_{i1}, \dots, \xi_{iL})^{\mathrm{T}}$ denote the mFPC score of the i-th subject in the elderly/young group, i = 1,…, N. Here, N =30 for the elderly group and N = 40 for the young group. The $\{\xi_i\}'s$ are partitioned by gender into $\{\xi_j^m\}$'s and $\{\xi_k^f\}$'s, where j =1, …, N_m and k = 1,…, N_f, for male and female subjects. Here (N_m, N_f) =(18,12) for the elderly and (N_m, N_f) =(16,24) for the young group. The null hypothesis we are testing is $H_0: E(\xi_j^m) = E(\xi_k^f)$ The bootstrap testing procedure is described as follows:

(a) Perform the mFPC analysis on the elderly/young group to obtain the mFPC scores $\{\hat{\xi}_j^m\}'s$ and $\{\hat{\xi}_k^f\}$'s for both male and female.

(b) Calibrate the scores so that the scores of male and female are of equal mean such that $\tilde{\xi}_j^m = \hat{\xi}_j^m - \bar{\xi}^m + \bar{\xi}$ and $\tilde{\xi}_k^f = \hat{\xi}_k^f - \bar{\xi}^f + \bar{\xi}$, where $\bar{\xi}^m$ and $\bar{\xi}^f$ are sample means of male and female and $\bar{\xi}$ is the pooled mean of $\{\hat{\xi}_i\} = \{\hat{\xi}_j^m \cup \hat{\xi}_k^f\}$.

(c) Resample $\{\tilde{\xi}_j^m\}$ and $\{\tilde{\xi}_k^f\}$ of size $N_m$and N_f, respectively, with replacement to form the bootstrap samples $\{\tilde{\xi}_j^{m,b}\}$ and $\{\tilde{\xi}_k^{f,b}\}$, b = 1 ,…,B. Here, we take B = 1000 for practical implementation.

(d) Calculate the test statistic T^2 =$\{1/N_m + 1/N_f\}^{-1}(\bar{\xi}^m - \bar{\xi}^f)^{\mathrm{T}}$, where D= diag$(\hat{\lambda}_1, \dots, \hat{\lambda}_L)$ and $\hat{\lambda}_l =$ var$(\hat{\xi}_{il})$, and the corresponding $\mathrm{T}^{2,b}$ for each bootstrap sample. Here, the off-diagonal entries of the covariance matrix are set as zero due to the fact ξ_{il} and ξ_{im} are unclrrelated for $l \neq$ m.

(e) Compute the p-value under the null hypothesis H_0 by p = $B^{-1} \sum_{b=1}^{B} 1(T^{2,b} > T^2)$

Table A: Results of ELDERLY group

	p-value		
	L=5	L=6	L=7
bootstrap<br>Hotelling's $T^2(\sim\chi^2(L))$	0.095<br>0.209	0116<br>0.161	0.158<br>0.155
FVE	84.5%	88.2%	90.7%

Table B: Results of YOUNG group

	p-value			
	L=5	L=6	L=7	L=8
bootstrap<br>Hotelling's $T^2(\sim\chi^2(L))$	0.099<br>0.271	0137<br>0.235	0.180<br>0.223	0.062<br>0.056
FVE	82.2%	85.4%	88.0%	90.2%
