# Supplementary material for: Heterogeneous Aging Effects on Functional Connectivity in Different Cortical Regions: A Resting-State Functional MRI Study Using Functional Data Analysis
Source: PLoS One. 2016 Sep 22;11(9):e0162028. doi: 10.1371/journal.pone.0162028 (PMC5033468; doi:10.1371/journal.pone.0162028)
Supplement: S2 File — (PDF) [file pone.0162028.s002.pdf]

Supporting Information

S2 File. ROI List of T1 Atlas

T1 Atlas ‘aparc.a2005s.annot’ was chosen from FreeSurfer (http://surfer.nmr.mgh.harvard.edu/), containing cortical anatomical label information of the whole brain with a total of 156 segmented regions. 120 of 156 cortical ROIs were selected from the union of ICA activation maps of the young and elderly groups. Removing limbic ROIs yielded 112 ROIs of the four cortical lobes. The ROIs in T1 Atlas ‘aparc.a2005s.annot’were listed in the following table and in the supplementary table of the manuscript.

number	T1 Atlas ‘aparc.a2005s.annot’<br>ROI name	hemisphere	
1	G_frontal_inf-Opercular_part	L,R	√
2	G_frontal_inf-Orbital_part	L,R	√
3	G_frontal_inf-Triangular_part	L,R	√
4	G_frontal_middle	L,R	√
5	G_frontal_superior	L,R	√
6	S_frontal_inferior	L,R	√
7	S_frontal_middle	L,R	√
8	S_frontomarginal	L,R	√
9	S_orbital-H_shapped	L,R	√
10	S_orbital_lateral	L,R	√
11	S_orbital_medial-Or_olfactory	L,R	√
12	S_suborbital	L,R	√
13	G_paracentral	L,R	√
14	G_parietal_inferior-Angular_part	L,R	√
15	G_parietal_inferior Supramarginal_part	L,R	√
16	G_parietal_superior	L,R	√
17	G_postcentral	L,R	√
18	G_precentral	L,R	√
19	G_precuneus	L,R	√
20	S_central	L,R	√
21	S_intermedius_primus-Jensen	L,R	√
22	S_intraparietal-and_Parietal_transverse	L,R	√

23	S_paracentral	L,R	√
24	S_parieto_occipital	L,R	√
25	S_postcentral	L,R	√
26	S_subparietal	L,R	√
27	G_temporal_middle	L,R	√
28	G_temp_supG_temp_transv_and_interm_S	L,R	√
29	G_temp_sup-Lateral_aspect	L,R	√
30	G_temp_sup-Planum_polare	L,R	√
31	G_temp_sup-Planum_tempolare	L,R	√
32	Lat_Fissure-ant_sgt-ramus_horizontal	L,R	√
33	Lat_Fissure-ant_sgt-ramus_vertical	L,R	√
34	Lat_Fissure-post_sgt	L,R	√
35	Pole_temporal	L,R	√
36	S_calcarine	L,R	√
37	S_temporal_inferior	L,R	√
38	S_temporal_superior	L,R	√
39	S_temporal_transverse	L,R	√
40	G_cuneus	L,R	√
41	G_and_S_occipital_inferior	L,R	√
42	G_occipital_middle	L,R	√
43	G_occipital_superior	L,R	√
44	G_occipit-temp_lat-Or_fusiform	L,R	√
45	G_occipit-temp_med-Lingual_part	L,R	√
46	Pole_occipital	L,R	√
47	S_collateral_transverse_post	L,R	√
48	S_occipital_anterior	L,R	√
49	S_occipital_middle_and_Lunatus	L,R	√
50	S_occipital_superior_and_transversalis	L,R	√
51	S_occipito-temporal_lateral	L,R	√
52	S_pericallosal	L,R	√
53	G_cingulate-Isthmus	L,R	x
54	G_cingulate-Main_part	L,R	x
55	G_insular_long	L,R	√
56	G_rectus	L,R	x
57	G_subcallosal	L,R	x
58	G_subcentral	L,R	√
59	S_subcentral_ant	L,R	√

60	S_subcentral_post	L,R	√
61	G_frontomarginal	L,R	xx
62	G_insular_short	L,R	xx
63	G_occipit-temp_med-Parahippocampal_part	L,R	xx
64	G_orbital	L,R	xx
65	G_temporal_inferior	L,R	xx
66	G_and_S_transverse_frontopolar	L,R	xx
67	Medial_wall	L,R	xx
68	S_central_insula	L,R	xx
69	S_cingulate-Main_part_and_Intracingulate	L,R	xx
70	S_cingulate-Marginalis_part	L,R	xx
71	S_circular_insula_anterior	L,R	xx
72	S_circular_insula_inferior	L,R	xx
73	S_circular_insula_superior	L,R	xx
74	S_collateral_transverse_ant	L,R	xx
75	S_frontal_superior	L,R	xx
76	S_occipito-temporal_medial_and_S_Lingual	L,R	xx
77	S_precentral-Inferior-part	L,R	xx
78	S_precentral-Superior-part	L,R	xx

(G:gyrus; S:sulcus; x: limbic region; xx: not ICA activated region)
